# Systematic review of the management of brain metastases from hormone receptor positive breast cancer

**DOI:** 10.1007/s11060-023-04276-9

**Published:** 2023-03-08

**Authors:** Shirley Jusino, Camilo E. Fadul, Patrick Dillon

**Affiliations:** 1 Ponce Health Sciences University, Ponce, PR 00716 USA; 2 Division of Neuro-Oncology, Department of Neurology, University of Virginia, Charlottesville, VA 22908 USA; 3 Division of Hematology/Oncology, University of Virginia, Charlottesville, VA 22908 USA

**Keywords:** Breast cancer, Brain metastases, HR +, Therapies

## Abstract

**Introduction:**

Brain metastases are a common cause of morbidity and mortality in patients with breast cancer. Local central nervous system (CNS) directed therapies are usually the first line treatment for breast cancer brain metastases (BCBM), but those must be followed by systemic therapies to achieve long-term benefit. Systemic therapy for hormone receptor (HR^+^) breast cancer has evolved in the last 10 years, but their role when brain metastases occur is uncertain.

**Methods:**

We performed a systematic review of the literature focused on management of HR^+^ BCBM by searching Medline/PubMed, EBSCO, and Cochrane databases. The PRISMA guidelines were used for systematic review.

**Results:**

Out of 807 articles identified, 98 fulfilled the inclusion criteria in their relevance to the management of HR^+^ BCBM.

**Conclusions:**

Similar to brain metastases from other neoplasms, local CNS directed therapies are the first line treatment for HR^+^ BCBM. Although the quality of evidence is low, after local therapies, our review supports the combination of targeted and endocrine therapies for both CNS and systemic management. Upon exhaustion of targeted/endocrine therapies, case series and retrospective reports suggest that certain chemotherapy agents are active against HR^+^ BCBM. Early phase clinical trials for HR^+^ BCBM are ongoing, but there is a need for prospective randomized trials to guide management and improve patients’ outcome.

**Supplementary Information:**

The online version contains supplementary material available at 10.1007/s11060-023-04276-9.

## Introduction

Breast cancer is the most commonly diagnosed cancer in women worldwide, with brain metastases being a major cause of morbidity and mortality [1]. It is estimated that 10–24% of metastatic breast cancers (MBC) seed the brain (30% per autopsy series) [2–4], and, in the United States, it is the second most frequent malignancy to cause brain metastases [5]. Approximately 7% of patients with MBC will have brain metastases at diagnosis (synchronous) while 17% will appear later on the course of the disease (metachronous) [6, 7]. Young age, lymph node positivity, and tumor characteristics (stage, grade, size, and Ki-67 index) correlate with higher incidence of breast cancer brain metastases (BCBM) [7–9]. In a recent meta-analysis, BCBM were found in 15% of patients with hormone receptor positive (HR^+^) and about 50% of HER2^+^ breast cancers [10].

Several prospective trials provide evidence to support management guidelines of HER2^+^ BCBM [11], but for patients with HR^+^/HER2^−^, the subtype with the highest absolute incidence of brain metastases, the evidence is scant and retrospective [12]. We performed a systematic review of the published data on approved and emerging systemic treatment options that could support their use for patients with HR^+^ BCBM.

## Methods

### Literature search

We conducted a systematic literature review according to the Preferred Reporting Items for Systematic Reviews and Meta-Analysis (PRISMA) guidelines [13]. We queried MEDLINE/PubMed and Cochrane Library for articles published between January 1964 and June 2022 using key terms to access clinical trials and original articles on current treatment options for HR^+^ BCBM. The search included combinations of the following keywords “HR^+^ breast cancer”, “ER^+^ breast cancer” “brain metastases”, “surgical resection”, “radiation therapy”, “systemic therapy”, “immunotherapy”, “chemotherapy”, and “targeted therapy”. The MEDLINE/PubMed, Cochrane Library, and EBSCO Essentials databases were searched on June 25, 2022. Abstracts and presentations from national meetings from 2019 to 2022 were also searched.

### Study inclusion and analysis

One author (SJ) screened all article abstracts and selected potential papers for inclusion. Another author (PD) determined if the selected papers met the inclusion/exclusion criteria. Studies were included if a primary or secondary analysis examined treatment safety or efficacy in HR^+^ BCBM. We excluded studies if they were not in English, were not peer reviewed, or were a letter or commentary article. Additionally, studies focused on leptomeningeal metastases were excluded. We included case reports, meta-analyses, reviews, and relevant retrospective and prospective studies that enrolled any BCBM participant with or without a pre-planned analysis of BCBM outcomes.

## Findings

**Fig. 1:**
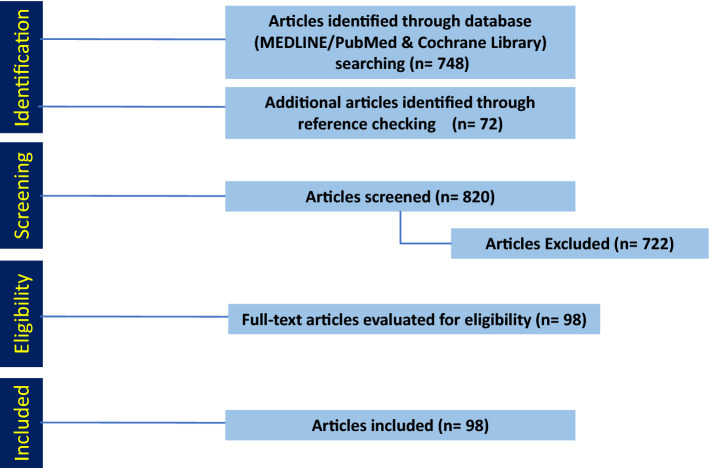
PRISMA diagram detailing the database search outcomes. A total of 748 articles were identified through database search. Another 72 additional articles were identified through references and added. Based on our inclusion and exclusion criteria 123 articles were included

The search in MEDLINE/PubMed and Cochrane Library yielded 748 articles that we screened for eligibility by title and abstract (Fig. 1). Additionally, we included 59 articles that we identified in the references. Of the 820 articles, we excluded 722 that did not meet the inclusion criteria, leaving 98 included in this systematic review.

## Discussion

### Local therapy for HR^+^ BCBM

The recommendations for local therapy (surgery and radiation) for HR^+^ BCBM are similar to those for brain metastases from other types of cancer and previously reviewed [14]. The use of local brain directed therapy depends on the patient’s functional status, the extent of systemic extra-neural disease, the number of metastases, the neurologic symptoms, and other comorbidities. Although there are no prospective randomized studies comparing surgery and stereotactic radiosurgery (SRS) for a single brain metastasis, surgical resection is considered when complete resection with low morbidity is feasible and when there is diagnostic uncertainty, bulky disease, high symptom burden, or when a very favorable extracranial disease profile exists. Resection followed by whole brain radiotherapy (WBRT) improved survival when compared to no adjuvant post-operative radiotherapy [15, 16]. A concern associated with WBRT is the long-term effect on neurocognitive function; thus, strategies to reduce the incidence include WBRT with hippocampal avoidance (HA) [17, 18] and memantine treatment [19].

Meanwhile, SRS is often the preferred approach to treat limited volume brain metastases. Metastatic volumes greater than 10 cm^3^ and progressive extra-cranial disease at the time of SRS were associated with worse survival for patients with BCBM [20]. Although the indication for SRS had previously been the presence of four brain metastases or less, recent guidelines from national societies suggest that some patients with more than four brain metastases may benefit from SRS [14, 21–23]. Moreover, SRS has the potential to reduce the risk of long-term radiation-induced neurocognitive impairment, while improving the quality of life [24]. In most instances, a case-by-case assessment by a multidisciplinary group with consideration of risk factors is the preferred approach.

### Systemic therapy

**Fig. 2:**
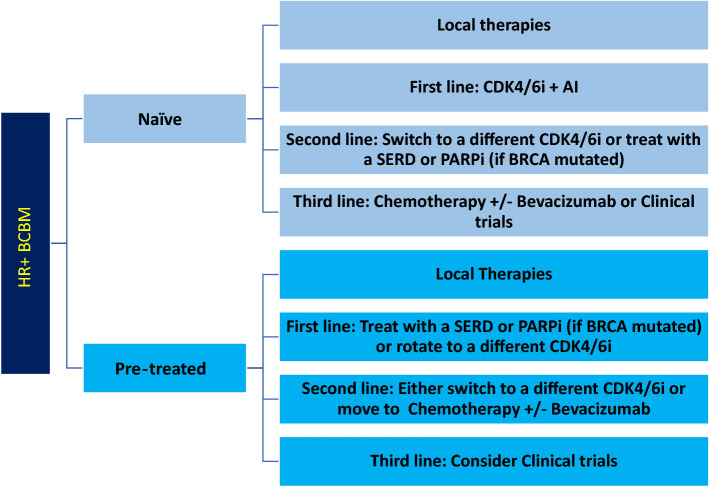
Suggested Line of Treatment for HR + BCBM. Local therapies (i.e., surgical resection and radiation) should be attempted first in naïve or pre-treated patients. Then, first, second-, and third-line systemic approach should be followed. *HR +* hormone receptor positive, *BCBM* breast cancer brain metastases, *CDK4/6i* cyclin dependent kinase 4/6 inhibitors, *AI* aromatase inhibitors, *SERD* selective estrogen receptor degraders, *PARPi* poly adenosine diphosphate-ribose polymerase inhibitors

After local therapies, patients with BCBM may benefit from systemic treatment due to the high frequency of additional recurrences both in the CNS and extra-neural. For HR^+^ BCBM, targeted therapy is preferred for first- and second-line systemic treatments, while cytotoxic chemotherapy is reserved for later lines of treatment or cases with refractory disease (Fig. 2).

#### Targeted therapy: CDK 4/6 inhibitors: palbociclib, ribociclib, and abemaciclib

Although the three FDA-approved CDK4/6i cross the blood–brain barrier (BBB), their clinical CNS efficacy is unproven. Palbociclib and abemaciclib are substrates of efflux transporters P-glycoprotein (P-gp) and breast cancer resistance protein (BCRP), while ribociclib is a substrate for P-gp [25]. Despite the limitations of CNS drug exposure from a pharmacologic standpoint, there are reports of clinical activity against HR^+^ BCBM.

Palbociclib was the first CDK4/6i approved for the treatment of HR^+^/HER2^−^ MBC with or without visceral metastasis based on two randomized clinical trials [26]. Both studies allowed patients with brain metastases, but only two and five patients were accrued, respectively (no data on CNS outcome is available). The information on treatment arm for patients who developed new brain metastases while on study was not disclosed.

Ribociclib was FDA-approved based on the results from the MONALEESA-2 [27, 28] and MONALEESA-3 [29] studies. The MONALEESA-2 study excluded patients with brain metastases [28]. In the MONALEESA-3 trial, eight of the 726 patients randomized (2:1) to receive ribociclib plus fulvestrant or placebo plus fulvestrant [30] had stable brain metastases, but no specific CNS outcome data is available.

Abemaciclib was FDA-approved following the results of a phase II single arm and two randomized clinical trials [31–33]. However, all three studies excluded patients with brain metastases. On the other hand, a single-arm phase II study evaluated the intracranial overall response rate (ORR) in HR^+^ BCBM brain or leptomeningeal metastases treated with abemaciclib [34]. The patients, grouped by tumor subtype, were treated with either abemaciclib or the standard of care therapy. Despite achieving excellent CSF drug concentration with an intracranial ORR of 5.2% and an intracranial clinical benefit rate (complete responses + partial responses + stable disease) of 24% in HR^+^/HER2^−^ patients, the study did not meet its primary endpoint of an intracranial ORR ≥ of 15%.

All three CDK4/6i have published case reports [35, 36] suggesting clinical activity, but there are no large controlled clinical trials demonstrating improved outcomes with these drugs for patients with HR^+^BCBM. Furthermore, there is scant information about appearance of new CNS metastases to draw conclusions about their ability to prevent development of BCBM. Based on their potential clinical activity and acceptable toxicity profile, an expert opinion suggested the use of CDK4/6i for patients with HR^+^ BCBM [37].

Data on re-treatment with CDK4/6i will be available as per the MAINTAIN clinical trial (clinicaltrials.gov NCT02632045), although this study excludes patients with active CNS metastases. Clinical trials with new CDK4/6i [dalpiciclib (NCT05586841)] and CDK2i [fadraciclib (NCT02552953)] are ongoing, but these studies exclude patients with active CNS metastases.

#### Endocrine therapy: tamoxifen, anastrozole, letrozole, and exemestane

Aromatase inhibitors (AI) are potentially active for the treatment of BCBM as they lower both serum and CSF concentrations of estradiol [38]. However, the only publications suggesting AI (or tamoxifen) have activity for HR^+^ BCBM are case series and reports [39–43]. Their potential efficacy is in the setting of BCBM naïve to endocrine therapies, but limited in tumors harboring ESR1 mutations or other endocrine resistance mechanisms [44]. A retrospective study of 198 patients with HR^+^ BCBM found that the median OS was significantly longer in patients who received endocrine therapy after a diagnosis of BCBM compared with patients who did not receive it (15 versus 4 months) [45]. Thus, for patients with newly diagnosed HR^+^ BCBM, it is reasonable to continue or start endocrine therapies in the setting of brain metastases, but combination therapy with a targeted agent is generally preferred.

#### Endocrine therapy: fulvestrant

Fulvestrant is the only FDA-approved selective estrogen receptor degrader (SERD) for breast cancer although several novel oral SERDs are in late-stage of development. Fulvestrant did not readily cross the intact BBB in animal studies [46] but two case series have suggested activity in patients with BCBM [47, 48]. The largest monotherapy fulvestrant study included patients with stable brain metastases, but outcomes for this specific group were not reported [49]. A phase II study [50] compared fulvestrant alone or in combination with capivasertib, an AKT inhibitor, in postmenopausal women with aromatase inhibitor-resistant HR^+^/HER2^−^ MBC, showing a significantly longer PFS of the combination over monotherapy (10.3 versus 4.8 months, *n* = 71). Although patients with BCBM were included, their outcomes were not reported. There are several ongoing trials using fulvestrant alone or in combination with novel agents, which allow inclusion of patients with BCBM (Table 1).

**Table 1:** Ongoing clinical trials for HR + BCBM

Identifier number	Title	Status
*Phase III*		
NCT02767661	A Phase III Randomized Controlled Study of Metronomic Capecitabine Combined with Aromatase Inhibitor Versus Aromatase Inhibitor Alone for First Line Treatment in Hormone Receptor positive, HER2 negative Metastatic Breast Cancer	Recruiting
NCT02437318	A Phase III Randomized Double-blind, Placebo Controlled Study of Alpelisib in Combination with Fulvestrant for Men and Postmenopausal Women with Hormone Receptor Positive, HER2 negative Advanced Breast Cancer Which Progressed on or After Aromatase Inhibitor Treatment	Active, not recruiting
NCT02947685	A Randomized, Open Label, Phase III Trial to Evaluate the Efficacy and Safety of Palbociclib + Anti-HER2 Therapy + Endocrine Therapy versus Anti-HER2 Therapy + Endocrine Therapy After Induction Treatment for Hormone Receptor Positive (HR^+^)/HER2 Positive (HER2^+^) Metastatic Breast Cancer	Active, not recruiting
*Phase II*		
NCT02738866	Phase II Trial of Palbociclib with Fulvestrant in Individuals with Hormone Receptor-Positive, HER2 Negative Metastatic Breast Cancer Who Have Progressed on Treatment with Palbociclib and an Aromatase Inhibitor	Recruiting
NCT02917005	A Phase II Study of Ovarian Function Suppression and Exemestane with or Without Palbociclib in PreMenopausal Women with ER Positive / HER2 Negative Metastatic Breast Cancer	Recruiting
NCT01441947	A Phase II Trial of Cabozantinib in Women with Metastatic Hormone-Receptor Positive Breast Cancer with Involvement of Bone	Active, not recruiting
NCT02632045	A randomized phase II trial of fulvestrant with or Without Ribociclib After Progression on Anti-estrogem Therapy Plus Cyclin-dependent Kinase 4/6 Inhibition in Patients with Unresectable or Metastatic Hormone Receptor^+^, HER2^−^ Breast Cancer (MAINTAIN Trial)	Active, not recruiting
NCT03051659	A Randomized Phase II Study of Eribulin Mesylate with Or Without Pembrolizumab for Metastatic Hormone Receptor Positive Breast Cancer	Active, not recruiting
NCT02675231	monarcHER: A Phase II, Randomized, Multicenter, 3-Arm, Open-Label Study to Compare the Efficacy of Abemaciclib Plus Trastuzumab with or Without Fulvestrant to Standard-of-Care Chemotherapy of Physician's Choice Plus Trastuzumab in Women With HR^+^, HER2^+^ Locally Advanced or Metastatic Breast Cancer	Active, not recruiting
*Phase Ib/II*		
NCT02983071	Phase I/II Safety, Pharmacokinetic, and Antitumor Activity Study of G1T38 in Combination with Fulvestrant in Patients with Hormone Receptor Positive, HER2 Negative Locally Advanced or Metastatic Breast Cancer After Endocrine Failure	Active, not recruiting
NCT04791384	Multicenter Open-Label Phase Ib/II Trial of Abemaciclib and Elacestrant in Patients with Brain Metastasis due to HR^+^/HER2^−^ Breast Cancer	Recruiting
NCT01872260	A Phase Ib/II, Multicenter Study of the Combination of LEE011 and BYL719 With Letrozole in Adult Patients with Advanced ER^+^ Breast Cancer	Active, not recruiting
NCT03054363	Phase Ib/II Open-label Single Arm Study to Evaluate Safety and Efficacy of Tucatinib in Combination with Palbociclib and Letrozole in Subjects with Hormone Receptor Positive and HER2 positive Metastatic Breast Cancer	Active, not recruiting
NCT02562118	Phase Ib Followed by Phase II Study of Pre-operative Treatment with Lenvatinib Combined with Letrozole in Post-menopausal Women with Newly Diagnosed Hormone Receptor Positive Breast Cancer with Measurable Primary Breast Tumor	Recruiting
NCT02871791	A Phase Ib/IIa Study of Palbociclib in Combination with Everolimus and Exemestane in Postmenopausal Women with Estrogen Receptor Positive and HER2 Negative Metastatic Breast Cancer	Active, not recruiting
*Phase I*		
NCT01791478	A Phase Ib Trial of BYL719 (an α-Specific PI3K Inhibitor) in Combination with Endocrine Therapy in Post-Menopausal Patients with Hormone Receptor-Positive Metastatic Breast Cancer	Active, not recruiting
NCT03099174	An Open Label, Phase Ib, Dose-escalation Study Evaluating the Safety and Tolerability of Xentuzumab and Abemaciclib in Patients with Locally Advanced or Metastatic Solid Tumours and in Combination with Endocrine Therapy in Patients with Locally Advanced or Metastatic Hormone Receptor-positive, HER2^−^, Breast Cancer, Followed by Expansion Cohorts	Active, not recruiting
NCT01273168	Phase I Trial of Z-Endoxifen in Adults with Refractory Hormone Receptor Positive Breast Cancer, Desmoid Tumors, Gynecologic Tumors, or Other Hormone Receptor-Positive Solid Tumors	Active, not recruiting
*Phase O & pilot studies*		
NCT04334330	Palbociclib, Trastuzumab, Pyrotinib and Fulvestrant Treatment in Patients with Brain Metastasis From ER/PR Positive, HER2 Positive Breast Cancer: A Multi-center, Prospective Study in China	Recruiting
NCT02942355	Pilot Trial of Anastrozole and Palbociclib as First-Line Therapy and as Maintenance Therapy After First Line Chemotherapy in Hormone Receptor Positive, HER2 Negative Postmenopausal Metastatic Breast Cancer	Active, not recruiting

#### Targeted therapy: PI3K/mTOR inhibitors

Everolimus is an mTOR inhibitor approved for late-stage HR^+^ MBC based on a randomized phase III trial (n = 724) [51] that suggested that the combination with exemestane offers a PFS benefit versus exemestane alone. While this study excluded BCBM, another phase II trial for BCBM, tested the CNS response rate to everolimus, trastuzumab, and vinorelbine [52] in HER2^+^ BCBM. The CNS response rate was 4%, the median intracranial time to progression was 3.9 months, and the median OS was 12.2 months, but the study did not meet its primary endpoint. A retrospective study of everolimus in patients with MBC and prior treatment observed a PFS of 6.8 months [53]. Nine patients with BCBM achieved a PFS of 6 months.

Alternatively, alpelisib may be an option in selected patients with PIK3CA mutations and brain metastases. Case reports (n = 4) [54] and a real world dataset with four additional cases (PFS of 43 days) [55] suggest that alpelisib may have CNS activity. Ongoing studies are examining either alpelisib or next-generation PI3K inhibitors in MBC and BCBM (NCT05230810).

#### Targeted therapy: PARP inhibitors

Olaparib, a PARP inhibitor with CNS penetration [56], has FDA approval in patients with MBC and a germline mutation in BRCA1 or BRCA2 genes. In an open-label phase III trial [57], monotherapy olaparib was compared with standard therapy in patients with a germline BRCA mutation and HER2^−^ MBC. The median PFS was significantly longer in the olaparib (7.0 months) than in the standard therapy group (4.2 months), but there were no significant differences in OS [58]. This study did not report on brain metastases. Another phase II study demonstrated that olaparib is an effective and tolerable treatment in patients with MBC (brain metastases allowed) and germline PALB2 or somatic BRCA1 and BRCA2 mutations [59]; there was no report of BCBM efficacy.

#### Targeted therapy: bevacizumab

Bevacizumab is a vascular endothelial growth factor (VEGF) inhibitor that improved PFS in patients with MBC treated in either the first-line or the second-line setting when combined with chemotherapy [60–64]. However, bevacizumab ultimately had no effect on OS and the FDA indication in breast cancer was rescinded in 2011. However, phase II clinical trials [65, 66] have shown that bevacizumab may be a reasonable option as an adjuvant to cytotoxic chemotherapy in BCBM.

#### Chemotherapy

Existing practice guidelines for treatment of MBC recommend sequential endocrine/targeted therapy until available agents have been exhausted before deploying systemic cytotoxic chemotherapy [67]. It is unclear if this recommendation applies to BCBM. Although cytotoxic agents may be faster acting against BCBM than certain targeted/endocrine therapies, it may be at the cost of greater toxicity. Several studies that report activity for cytotoxic agents against BCBM fail to describe cohort characteristics including receptor status, undermining the establishment of their efficacy among the distinct breast cancer subtypes [68–72].

Capecitabine is often the first chemotherapy attempted for HR^+^ BCBM [10, 73], because it is thought to penetrate the BBB [74]. A retrospective study [75] and a phase I trial [69] reported responses in the brain. Likewise, methotrexate penetrates the BBB and exhibited PR (28%) responses in a retrospective study [71]. A non-randomized prospective study reported that treatment with the CMF (cyclophosphamide, methotrexate, and fluorouracil) or FAC (5-fluorouracil, doxorubicin, and cyclophosphamide) regimens led to a 59% CNS response [76]. Furthermore, a prospective study (*n* = 56) revealed that cisplatin and etoposide resulted in CNS response, including seven CR, 14 PR, and 12 SD [77]. Other drugs that cross the BBB and have reported clinical data include temozolomide [78], doxil [79], eribulin [80] and irinotecan [81].

### Combination local and systemic therapy

The combination of chemotherapy and radiation may have synergistic effect against brain metastases. A prospective study compared the efficacy and impact on the quality of life of WBRT and chemotherapy in patients with BCBM [81]. This study randomized 58 patients stratified according to breast cancer subtypes to receive WBRT alone or WBRT plus carboplatin. The ORR was 34.4% for WBRT alone and 79.3% when combined with cisplatin. The OS (15.9 versus 11.3 months) and the PFS (10.2 versus 6.8 months) were significantly longer in the WBRT plus chemotherapy group when compared to the WBRT cohort. Karnofsky Performance Status scores significantly improved after WBRT plus chemotherapy compared to WBRT alone, while the combination had similar adverse reactions.

A phase I trial showed that bevacizumab combined with WBRT was safe and generated response in patients with brain metastases from solid tumors (n = 19), including breast cancer (n = 13) [82]. There was an 87.5% response rate at the highest dosing level (WBRT 30 Gy in 10 fractions and bevacizumab 15 mg/kg on days 1, 15, and 29).

Specifically, for patients with HR^+^ BCBM, a retrospective study of concurrent radiotherapy with CDK4/6i, palbociclib (n = 34) or abemaciclib (n = 2), resulted in brain metastases local control at 12 weeks of 91.7% [83]. This outcome is provocative but there is need for prospective controlled studies to support any recommendation on the combination of radiation and CDK4/6i for patients with HR^+^ BCBM.

### Emerging therapies

#### Immunotherapy/antibody–drug conjugates

Immunotherapy is not approved for metastatic HR^+^ breast cancer (aside from rare patients with high tumor mutational burden or mismatch repair deficient cancers). A phase II (NCT02886585) study is evaluating the safety and efficacy of pembrolizumab, a checkpoint inhibitor (PD-1), in CNS metastases (brain and leptomeningeal) from multiple tumors (including breast cancer). Preliminary results from this study suggest efficacy of pembrolizumab in the treatment of leptomeningeal disease from solid tumor malignancies (n = 20, including 7 HR^+^/HER2- and 3 HR + /HER2^+^) [84], but results pertaining to brain metastases have yet to be published.

Recent phase I and II studies have shown positive results with trastuzumab deruxtecan, an antibody–drug conjugate linked to a topoisomerase I inhibitor in patients with HER2^low^ MBC [85, 86]. A phase III trial [87] evaluated the efficacy and safety of trastuzumab deruxtecan (n = 373, HR^+^ = 331) in HER2^low^ MBC patients compared to physician’s choice of chemotherapy (eribulin, capecitabine, paclitaxel, or gemcitabine) (n = 184, HR^+^ = 163). Trastuzumab deruxtecan significantly prolonged median PFS (10.1 versus 5.4 months) and OS (23.9 versus 17.5 months) when compared to the control arm. In the trastuzumab deruxtecan and the chemotherapy cohorts, 5.4% and 4.3% of patients had brain metastases. The brain metastases ORR was 67.4% [88] suggesting that trastuzumab deruxtecan has activity in patients with HR^+^, HER2^low^ CNS metastases.

#### New compounds

Sacituzmab govitecan and Elacestrant received indications in HR + breast cancer in 2023 and will be studied for activity in HR + BCBM (no CNS efficacy data available to date). Multiple drugs with potential efficacy in HR^+^ BCBM are being studied in preclinical and clinical studies. A highlight is ANG1005, which consist of three paclitaxel molecules covalently linked to Angiopep-2 and crosses the BBB via the LRP1 (low-density lipoprotein receptor-related protein 1) transport system [89]. An open-label phase II study in BCBM (n = 72, 39 HR^+^) revealed an 8% intracranial ORR, better for patients with HER2^+^ (14%) than those with HER2^−^ (3%).

Another phase I study [90] evaluated the optimal dose for an AKT inhibitor (MK-2206) administered in combination with anastrozole, fulvestrant, or both in postmenopausal women with HR^+^/HER2^−^ MBC (n = 30). Nineteen patients had visceral involvement (including brain metastases). Preliminary results showed PR in 7.7% of the patients and a CBR of 36.7% and ORR rate of 15.4%. The most common adverse events were rash (33.3%), hyperglycemia (20%), hypophosphatemia (16.7%), and fatigue (10%).

## Recommendations

There is no level 1 evidence based on prospective randomized clinical trials to provide guidance on systemic therapies for HR^+^ BCBM. The current potentially effective first-line systemic therapies for HR^+^ BCBM, (Fig. 2) include CDK4/6i (palbociclib, ribociclib, or abemaciclib) in combination with aromatase inhibitors, or SERDs. Potential options for second-line systemic treatments include trastuzumab deruxtecan if HER2^low^, CDK4/6i rotation, a mTORC1 inhibitor, a PARP inhibitor if BRCA mutated, or other molecularly targeted inhibitors such as alpelisib (usually given with an endocrine agent). Pre-treated patients may have endocrine resistance (i.e., ESR1 mutation), thus, a personalized approach based on molecular testing may be of benefit. Upon exhaustion of targeted/endocrine therapies, chemotherapy agents such as capecitabine, trastuzumab deruxtecan, eribulin or others (with or without bevacizumab) could be an option (Table 2).

**Table 2:** Systemic agents publicly reported in HR^+^ BCBM and best available efficacy data

Agent(s)	Ref	Patients with HR^+^HER2^−^ BCBM	PFS in HR^+^HER2^−^BCBM (months)	ORR/CBR in BCBM	OS (months)	Level of evidence	Notes
CDK4/6i + XRT	83	36	–	91.7%	–	4	Retrospective series
Capecitabine	75	7	–	85% CBR		2b	3 of 7 with CR
Bevacizumab, cisplatin, etoposide	66	6	3.7	83%	4.8	2b	No CR’s. OS lowest in HR^+^HER2^−^
Trastuzumab Deruxtecan	87	18	–	67.4%	23.9	1b	HR^+^HER2^−low^ subset data. From a RCT vs chemo. N = 557 total
Cytoxan, methotrexate, 5FU (CMF)	76	20*	–	59%	6	2b	Published in 1992. Single arm prospective
Bevacizumab + carboplatin	65	9	5.62	56%	14.10	2b	Subset from the HR^+^HER2^−^ cohort
Cisplatin, etoposide	77	59*		38%	7.5	2b	Published 1999
Methotrexate 3.5 g/m2	71	18	–	28%	4.5	2b	9 patients had leptomeningeal component
Carboplatin + WBRT	81	25	10.2	34.4% ORR	15.9	2b	Randomized. Mixed receptor status
Abemaciclib + E	34	58	4.9	24% CBR	12.5	1b	OS = 12.5 months. Prospective trial. Did not meet 1° endpoint
Alpelisib + E	54	4	5 (mean)	(4 of 4 w response)	–	4	PFS of 3, 5, 6, 6 months
Eribulin	80	22	5.0	14% ORR 48% CBR	7.0	2b	Prospective observation study, 6 sites. 65% HR^+^
Doxil + cytoxan	79	20	3.6	41%ORR	23	4	Retrospective single center experience
Everolimus + E	53	7	18.2	–	–	2b	Subset analysis
Ribociclib + E	30	8	–	–	–	2b	SD in 8 patients w HR + BCBM
AI, tamoxifen or fulvestrant after local therapy	45	88	–	–	15	4	Retrospective comparison to local treatment alone
Fulvestrant	48	3	25	(3 of 3 w response)	35	4	Mean of 3 patients

Ordered by highest CNS overall response rate (ORR) and highest number of patients reported with HR^+^HER2^−^BCBM (single case reports excluded; missing outcomes excluded). Eligibility requirements, assessments, and accruals vary widely across reports; cross-trial comparisons are not reliable. Note that insufficient data are available for two of the CDK4/6 inhibitors (without radiation) and olaparib. VEGF inhibitor studies may artificially improve tumor measurements and should be interpreted with caution. Note that targeted agents were combined with endocrine agents (E) as per FDA approvals. Level of evidence as per AHRQ guidelines

*RCT* randomized clinical trial, *XRT* radiation

*Prior to the era of HER2 testing

Expert opinions/recommendation in the area of HR^+^BCBM are limited since many published studies fail to disclose the receptor status or to make direct correlations between receptor status, brain metastases, and treatment response. Furthermore, at least 20% of BCBM have receptors that differ from the primary cancer [91–97].

## Conclusion

Despite the advances in systemic therapies for HR^+^ breast cancer, the treatment of brain metastases remains a major therapeutic challenge that requires a multidisciplinary approach. The contemporary recommendations for the treatment of HR^+^ BCBM involve local therapies; maximal local control with surgery, SRS and WBRT with the option of repeated local therapy for recurrence whenever feasible [14].

Clinical trials are increasingly available for patients with BCBM (Table 1), but the field needs randomized clinical trials of new drug candidates that include patients with BCBM and report separately on their outcomes. Research into distinct biomarkers BCBM that could aid in early detection and improve personalized targeted therapy is needed. Screening for brain metastases in patients with MBC is not generally recommended; however, approximately 20% [98] of patients with BCBM are asymptomatic. Asymptomatic patients have less CNS metastatic burden and better outcomes than patients who are symptomatic [99]. Noninvasive techniques such as liquid biopsy presents an emerging aspect of breast cancer care that may help improve future CNS surveillance.

Survival from HR^+^ breast cancer is improving as drugs that are more effective become available, but as patients with MBC live longer, the likelihood of CNS relapse increases. The recommendations for local therapies are robust, but systemic therapy recommendation are limited by the quality of evidence. There is urgency to study new and potentially more effective therapies in well-designed, clinical trials to improve outcomes of the growing population with breast cancer and brain metastases.

## Supplementary Information

Below is the link to the electronic supplementary material.

Supplementary file1 (DOCX 31 KB)

## Data Availability

Not applicable
